# Measuring the Welfare Impact of Soft-Catch Leg-Hold Trapping for Feral Cats on Non-Target By-Catch

**DOI:** 10.3390/ani9050217

**Published:** 2019-05-05

**Authors:** Chantal Surtees, Michael C. Calver, Peter R. Mawson

**Affiliations:** 1 Environment and Conservation Cluster, School of Veterinary and Life Sciences, Murdoch University, Murdoch, WA 6150, Australia; M.Calver@murdoch.edu.au; 2 Perth Zoo Science, Department of Biodiversity, Conservation and Attractions, South Perth, WA 6151, Australia; Peter.Mawson@dbca.wa.gov.au

**Keywords:** animal, welfare, by-catch, cat, trapping

## Abstract

**Simple Summary:**

Feral cats are linked to fauna extinctions and declines globally through predation, disease transmission, and hybridisation. Soft-catch leg-hold trapping, with or without olfactory lures, is used to capture feral cats; however, by-catch may occur, including species of conservation concern. Using data from 431 non-target animals trapped at six Western Australian sites over 18 years, we demonstrate that birds are at greatest risk of serious injury. Appropriate placement of traps, careful choice of lures depending on the non-target species known to be in the study area, adjustment of traps to minimize the chance of closing on small animals, and training of all personnel associated with trapping will minimize the risk to birds and other non-target fauna.

**Abstract:**

To inform trapping protocols to reduce by-catch while trapping feral cats by-catch welfare costs should be quantified. During cat trapping programs at six Western Australian sites from 1997–2015, 431 non-target individuals, including 232 individuals from native species (132 mammals, 52 birds and 42 reptiles) were captured. Among the native fauna; birds were more likely to be severely injured (33%, compared to 12% in mammals and 21% in reptiles). Amongst other vertebrates, larger individuals were less likely to be injured. Olfactory lures used in these studies attracted reptiles, but repelled mammals. By-catch varied with climate and landscape. Trap injury to by-catch species poses ethical concerns, especially for threatened species that can least afford an additional threat. Future trapping should consider the timing of trapping, trap placement, trap settings (especially the treadle pressure needed to close the trap) and new innovations sending immediate capture alerts to minimise by-catch and potential injury associated with prolonged restraint. By-catch welfare data should be analysed to identify best practice and on-going improvement.

## 1. Introduction

Humans have spread the domestic cat *Felis catus* globally, where its feral populations frequently have devastating effects on native fauna through predation, spread of disease, and hybridisation with native felids. This has caused declines or local extinctions of island [1,2,3] and mainland fauna [4,5,6]. Overall, together with introduced rodents, the cat has contributed to 44% of modern reptile, bird and mammal extinctions [7] and is a threat to numerous other terrestrial vertebrates [8]. For example, under the provisions of the Australian *Endangered Species Protection Act 1992* (superseded by the *Environmental Protection and Biodiversity Conservation Act 1999*), predation by feral cats is listed as a Key Threatening Process [9]. Feral cats now threaten 142 species and subspecies of Australian fauna [10], with direct evidence of predation on three critically endangered, five endangered, eight vulnerable, and 12 near-threatened species [7]. Hence control of feral cats is critical for conservation management, and the current methods are trapping, shooting and baiting.

Trapping, as a control method, offers opportunities to catch and remove individual problem cats or to trap them for tagging and releasing as part of ecological studies. There is a long history involving the use of leg-hold traps to seize a pest animal by a limb, after which it is shot when the traps are cleared. At first steel jaw snap traps were used, but they are now regarded as inhumane. The injuries sustained by the individuals caught, by-catch included, are so serious (e.g., Tullar 1984 [11]) that the use of these traps is now specifically regulated or banned in 108 jurisdictions globally [12]. For example, in the United States, nine states restrict the use of leg-hold traps, with New Jersey’s legislation stating ‘No person shall manufacture, sell, offer for sale, possess, import or transport an animal trap of the steel-jaw leghold type’ (NJSA 23:4-20 to 23:22.8, available online at https://www.animallaw.info/statute/nj-fur-unlawful-trapping-article-2-manner-means-and-times-hunting#s221). Therefore, a modified version of this trap type was necessary to reduce injury and suffering to target animals before they were shot, and to reduce injury to non-target species so that they could be released. Traps with padded jaws were trialed, leading eventually to the final product of the soft-catch leg-hold trap [11,13,14,15].

Soft-catch leg-hold trapping is used to capture feral cats with recorded injuries to the target animals mainly limited to minor bruising and abrasions [13,14,15,16]. However, even the use of soft-catch leg-hold traps is restricted or prohibited in some jurisdictions. For example, within Australia, not all states and territories permit leg-hold traps for the control of feral cats [17]; Tasmania, for instance, does not allow any leg-hold trapping unless an application to the Minister has been approved as described in the Tasmanian *Animal Welfare Act 1993* [18]. As a result, trapping of cats in these areas is limited to cages [17], despite experience indicating that feral cats in remote areas avoid cage traps but can be trapped with leg-hold traps, while even where cage traps are effective they are biased to younger, female cats [19] and that cage traps and leg-hold traps can be equally effective in catching cats in agricultural settings [20].

One possible objection to the use of soft-catch leg-hold traps is that the ‘trap safety’ of soft-catch leg-hold traps in regard to by-catch is not as well-researched as the effect on by-catch associated with other methods of capture/control such as shooting or baiting [13,14,15,21,22]. Given that the native fauna potentially being captured by these traps are often much smaller than the cats targeted in the trapping programs, any injuries sustained can range from a simple, mild oedema (swelling) [11] to death [16]. Durham [23] suggests that this variability is due to the difference in anatomy between various taxa. For example, unlike mammals, birds have minimal soft tissue in the leg to cushion injury to blood vessels, nerves and bones. Injury identification itself presents difficulties; not all injuries are obvious and visible externally [23]. Additionally, some injuries will have a greater impact on the survivorship of the individual than first anticipated; for example, a raptor with an injured leg will have difficulty in hunting, thus decreasing survivorship by risking starvation [23].

Here, we document injuries to non-target species caused by soft-catch leg-hold traps using by-catch records from six locations across Western Australia where these traps were used to capture feral cats. The sites were not equal in feral cat density. We also determined the influence of olfactory lures on the relative proportions of by-catch of different taxa; the relative proportions of native and non-native by-catch; and, if mass and taxa affected the severity of injury to native by-catch. We offer recommendations for reducing the incidence of by-catch when trapping for feral cats using soft-jaw leg-hold traps and areas for further research.

## 2. Materials and Methods

### 2.1. Data Sources

Researchers overseeing trapping programs using soft-catch leg-hold traps (Victor^®^ Soft-catch traps; #1.5 and #3) at six sites in Western Australia between 1997 and 2015 kindly agreed to share their data on by-catch from their programs. The sites ranged in location from the Kimberley Region to the South West Region (Table 1). The trapping programs were conducted with differing purposes, with no standardisation in the field data recorded nor in the skill of the trappers.

### 2.2. Trapping Protocols

Trapping protocols varied. At Mt Gibson Sanctuary traps were placed within a fenced reserve, while at Perup Sanctuary they were placed both inside and outside a fenced reserve. At Francois Peron National Park, Boyicup, Balban and Mornington Sanctuary traps were placed in a large, unfenced area. The use of attractants and trap sets also varied across sites. At three sites records were kept of the type of a lure or attractant that was used; FAP (felid attracting phonic electronic device; [24]) and PONGO (a mixture of feline faeces and urine) were both used at Perup Sanctuary, a combination of fresh urine and faeces from a domestic cat and local soil, along with a visual attractant at the back of a bower (a covered recess made of vegetation) were used at Mornington Sanctuary, and 29 combinations of lures and sets were used at Francois Peron National Park.

Additionally, the trapping effort and the area covered varied across sites. Perup Sanctuary had only 10 nights of trapping whereas the others had considerably more; Boyicup had 409, Balban had 669, Mornington Sanctuary had 1112 and Mt Gibson Sanctuary had 4762. The total number of trapping nights was not recorded for Francois Peron National Park, but trapping was conducted from 1997–2005. Trapping effort could not be calculated due to a lack of data regarding the number of traps used for each trapping event.

### 2.3. Data Management

The data were pooled across all the sites and each by-catch trap event was assigned to an injury category (Table 2) based on a scale described in [11], which is an adaptation of the categories originally developed by Van Ballenberghe [25].

Category 0 was added to account for uninjured by-catch. Further modifications included: when the individual was initially released unharmed and injuries were later observed, it was assigned category 0 as the injuries cannot be guaranteed as a result of trapping; if an injury was recorded but no supporting data provided then the entry was considered an error and category 0 was assigned to the individual; abrasions were considered a laceration of less than 25 mm; and any individual that died or required euthanasia due to injuries sustained was assigned category 4. If multiple injuries were sustained by the individual, the categorization was allocated based on the most severe of these injuries.

In some cases, the mass of non-target animals was recorded at the time of trapping. When they were not, the steps set out in Table 3 were applied to assign a mass. The species’ mass was taken from [26,27] for mammals; [28,29] for birds; and [30] and Mike Bamford (pers. comm., bamford.consulting@iinet.net.au) for reptiles. Estimates of mass applied to 87% of all records because masses were only recorded for a subset of by-catch at the Francis Peron site. We acknowledge that this might introduce bias, but the alternative is to attempt no assessment of the importance of mass at all.

All animals captured were described as either target (cats) or non-target (all other individuals captured) regardless of the original study’s purpose. Non-target species were further categorised as native or non-native. Lures and attractants were classified as either ‘olfactory’ or ‘other’. ‘Other’ lures included all those that did not primarily stimulate taste or smell. 

### 2.4. Statistical Analyses

The data were pooled across all the sites and each by-catch trap event was assigned to an injury category (Table 2) based on a scale described in [11], which is an adaptation of the categories developed earlier [25]. Statistical analyses were carried out using online routines in VassarStats (http://vassarstats.net), Microsoft Excel, and StatSoft, Inc. (2006) STATISTICA (data analysis software system, Version 7.1; www.statsoft.com).

Chi-square contingency tables were used to assess the association between the injury categories of the native fauna by-catch and the taxa sustaining them. To ensure that minimum expected values were met for a valid test, the analysis combined the number of individuals that had no injuries (uninjured), had an injury ranging from category 1 to 3 (intermediate), or had category 4 (severe) injuries, resulting in a total of 3 levels of injury [31]. Percentage deviations (a measure of the extent to which the observed chi-square value varies from that expected if there is no association between the variables) were also calculated.

ANOVA was used to investigate the effect that mass had on the severity of injuries sustained for each taxon separately; the three injury categories as described for the chi-square test (independent) and the log_10_ value of individual mass. Log-transformation of the mass was necessary due to the wide, unequal mass ranges in different taxa.

### 2.5. Ethical Approvals

Animal ethics approval was provided for the projects located at: Mornington Sanctuary (University of Tasmania Animal Ethics Committee A0011661, and DEC AEC 2013-40); Francois Peron National Park (CALM AEC 1996-09 and 2003-35) and Perup Sanctuary (DEC AEC 2012-41).

## 3. Results

### 3.1. By-Catch

A total of 431 non-target individuals, including 232 individuals from native species, were captured across the six sampling sites (Table 4). Non-target species that are also declared pests in Australia (e.g., red foxes *Vulpes vulpes* and the European rabbit *Oryctolagus cuniculus*) are included in Table 4 and are counted as non-target species in all subsequent analyses.

### 3.2. Lures

There was a significant association between the type of lure used to attract the target species and the taxon of the non-target individuals captured ($\chi_{2}^{2}$ = 9.95, *p* = 0.007). Reptiles were more likely to be captured with olfactory lures than mammals or birds, with mammals being the least likely to be captured (Table 5).

### 3.3. Injuries

To compare injuries between native and non-native fauna in by-catch, data were combined to form the categories uninjured, intermediate injury (combining categories 1, 2 and 3 together; animals survived) and category 4 (injuries were severe and often resulted in death) (Table 6). Intermediate and severe injuries occurred significantly more frequently amongst native fauna ($\chi_{2}^{2}$ = 14.22, *p* = 0.0008) (Table 6).

Within the 232 animals trapped as native by-catch, 65 individuals were injured or died (Table 7). The fate of an individual was associated significantly with the taxon it belonged to ($\chi_{4}^{2}$ = 11.31, *p* = 0.023). Mammals had the highest rate of individuals being unharmed, whereas the birds incurred the largest proportion of category 4 injuries and were far more likely to be killed (% deviation = +76.4) than any other taxon (Table 7). Overall, any given individual bird was likely to be unharmed, however when injury did occur, it was mostly severe (category 4), with some resulting in death (Table 7).

Analysis of the relationship between taxa, mass and injury showed that for birds, body mass did influence the likelihood of injury (F_(2,48)_ = 12.00, *p* = 0.006). Post-hoc Tukey’s tests (unequal sample sizes) showed a difference in mass between uninjured and severely injured birds (*p* = 0.0004), with examination of the means showing that lighter animals are more likely to be severely injured (Table 8). Injuries among mammals were also influenced by body mass (F_(2,355)_ = 6.13, *p* = 0.002). Post-hoc Tukey’s tests (unequal sample sizes) also showed a difference in mass between uninjured and severely injured mammals (*p* = 0.026) and between moderately injured and severely injured mammals (*p* = 0.019). Examination of the means also showed that lighter animals were more likely to be severely injured (Table 8). Mass did not influence the likelihood of injury to reptiles (F_(2,37)_ = 6.13, *p* = 0.194).

## 4. Discussion

### 4.1. By-Catch

Snap traps, specifically soft-catch traps, such as the ones used throughout this project, have a reputation for trapping by-catch [16]. Their efficiency is highly variable, depending on a variety of factors such as pan spring tension, environmental conditions, target and non-target species’ population demographics, trapper expertise, seasonal variation, site characteristics and previous exposure of the targeted individuals to trapping [16,32,33].

The soft-catch leg-hold traps captured more by-catch than targeted individuals at four out of the six sites. At both the Mornington Sanctuary and Francois Peron National Park sites considerably more target than non-target species were captured; with the latter capturing almost 10 times as many targeted individuals than by-catch. The relative proportions of target and non-target species trapped is likely the result of many interacting factors, including the number of target species at the study site, the non-target species present and the skill in setting traps.

### 4.2. Lures

Using attractants at trap sites can provoke varied responses amongst wild animals, both target and by-catch [34]. The response of the individual animal depends on prior knowledge (past experience, acquired from other individuals, or inherited), environmental cues, or its physiological condition and social status [34]. The lures used at Francois Peron National Park; olfactory (stimulating the sense of smell) or otherwise, showed a definite relationship with the taxa of by-catch. Birds were indifferent to either lure type, while olfactory lures repelled the mammals and attracted the reptiles. The effect that olfactory lures had on the response of reptiles and mammals can be a result of several factors.

The scat avoidance hypothesis explains the avoidance of prey animals to their predators’ faeces and provides a potential means to reduce predation risk [35]. This aversion towards predator scents has been identified in several mammalian species [34]. As feral cats are known predators of all the mammals present in this study, it is reasonable to deduce that potential mammalian by-catch could actively avoid the PONGO laced traps.

Reptiles, on the other hand, are attracted to strong odours and pungent baits [36]. All but one olfactory attractant used at Francois Peron National Park utilised canine or feline PONGO. When considering this effect, the higher by-catch rate of reptiles can be expected. Fortunately, reptile by-catch are less likely to be severely injured than birds, but this ‘benefit’ could be offset by the effects of meso-predator release of larger reptiles (e.g., *Varanus* spp.) that has been observed following the control of foxes and feral cats [37], if the increased reptile population size results in a corresponding increase in by-catch capture rate.

### 4.3. Injuries

We found that intermediate and severe injuries occurred far more often (Table 6) amongst the native fauna than the non-native. This is likely because the non-native species were quadrapedal and often heavier than the native species. When quadrapedes are trapped they still have three legs free to manoeuvre into a position that doesn’t exacerbate minor injuries. They were often heavier too (excluding rabbits), which reduced the chance of severe injury.

Birds were most likely to be severely injured when trapped. This could be attributed to their skeleton consisting of long, thin walled, hollow bones [38] unlikely to withstand the snapping pressure of a trap. Birds also have less muscle mass (compared to mammals). Therefore, there is less cushioning to the bones, nerves and blood vessels, as well as a reduced vascular supply to their extremities; meaning that when an injury does occur, they have a decreased ability to heal or fight infection resulting in the likelihood of the limb seizing, regardless of immediate visible injury [23]. This delayed effect can also affect survivorship by reducing hunting ability (particularly in predatory birds) or leaving the individual vulnerable to predation due to a decreased ability to escape [23]. Thus, when an injury is visible, the full extent of the impact may not be immediately recognised and result in a delayed death of the individual; this was not investigated in this project as injury assessment was conducted immediately before release from the trap only. Consequently, the extent of injury and negative impact on the bird life captured throughout this study could be underestimated.

The mass of the individual also influenced the severity of injury for birds and mammals, with those that were lighter being more likely to be severely injured. This trend was noted by Powell et al. [39], who stated that by-catch, particularly of non-target animals smaller than the target, can potentially suffer severe injuries. Seddon et al. (1999 in [40]) were forced to modify their trapping techniques for cats when the much smaller burrowing bettong (*Bettongia lesueur*) was present, in an effort to reduce the chance of “excessive injury” being sustained.

For a leg-hold trap to clamp shut, the trigger must be released. This is only possible if the individual captured either equals or exceeds a particular mass (usually that of the target species; [39]). Powell et al. [39] claim that by correctly matching the size of the leg-hold trap to the target species and using the correct set, injury can be minimised and the rate of by-catch reduced, respectively. However, if the trigger plate of the trap has a low tripping force or a smaller-than-target-species individual applies excess force (mass × velocity) to the trigger plate (i.e., by jumping or falling on it), the trap jaws will clamp shut. This can be seen in smaller bipedal animals such as hopping macropods, putting them at risk of landing on the trigger plate with exaggerated force and triggering a trap despite their smaller mass.

### 4.4. Recommendations

In the interests of animal welfare and the conservation of non-target species, we recommend that oversight of future feral cat leg-hold trapping practices focus on both the target species and the potential by-catch in the study site, especially regarding the morphology, behaviour and conservation status of the potential by-catch species. The question of whether or not to use leg-hold traps in preference to cage traps should also be considered. Ethically, cage traps pose less risk to by-catch species and may also cause less stress to by-catch and cats before traps are cleared. However, cage traps are less effective when cats have little or no prior exposure to people; even when they do catch cats the catch is biased to juveniles and females [19].

When the decision to use leg-hold traps is justified, potential measures to reduce by-catch and increase the safety of by-catch include:

1. Ensuring that the triggers for traps are set to respond to the mass of the target species and no lower, to reduce the chance of catching smaller, lighter fauna more likely to be injured.
2. Raised sets (traps placed on a platform above ground level) can be used to reduce the occurrence of trapping ground dwelling by-catch.
3. Given that large, predatory reptiles such as varanids are attracted to strong odours, visual lures should be considered as an alternative to olfactory lures when reptile by-catch is likely.
4. Trappers should be fully trained in the correct use and placement of the traps based on current trapping standards, as well as the correct handling and injury assessment of both the target and by-catch species. This should reduce the occurrence of by-catch and severity of injury to those individuals while under manual restraint, as well as ensuring the correct course of action if a non-target animal is injured.
5. Implementation of an effective Environmental Management System (EMS) to evaluate trapping programs, including non-target impacts.
6. Careful cost-benefit assessments if there are potential risks to non-target species of conservation concern to ensure that a program designed to protect these species does not endanger them.
7. Good field records be kept of the type of by-catch and the injuries observed so that practice can be constantly reviewed and improved.

## Figures and Tables

**Table 1:** The location details of the six study sites from which cat and by-catch trapping data were sourced.

Region	Location	Co-Ordinates	Climate
South-West	Boyicup Forest	−34.28 S, 116.57 E	Mediterranean
	Balban Forest	−34.09 S, 116.35 E	Mediterranean
	Perup Sanctuary	−34.25 S, 116.14 E	Mediterranean
Gascoyne	Francois Peron National Park	−25.84 S, 113.56 E	Semi-desert Mediterranean
Kimberley	Mornington Sanctuary	−17.51 S, 126.12 E	Tropical Monsoon
Avon Wheatbelt	Mount Gibson Sanctuary	−29.61 S, 117.12 E	Semi-arid warm Mediterranean

**Table 2:** Categorisation of injury types for comparison of injuries associated with soft-catch leg-hold traps.

Category	Injury Description
0	Uninjured.
1	Slight foot/leg oedema, no lacerations or broken bones.
2	Moderate oedema, compressed muscle, lacerations less than 25 mm long, no broken bones and joints.
3	Lacerations at least 25 mm long, visible tissue damage, no tendon damage, one metacarpal or phalanx bone broken.
4	Combinations of deep, wide lacerations, severed tendons, broken metacarpals, broken radius or ulna bones, broken ankles or legs and joint dislocations.

**Table 3:** Criteria used for body mass determination of by-catch. (Note: There was no occurrence of sex being recorded without age).

	Sex		
Age	Female	Male	Not Recorded
Juvenile	Minimum mass range for females	Minimum mass range for males	Average of the female and male minimum mass range
Adult/ Sub-Adult	Maximum mass range for females	Maximum mass range for males	Average of the female and male maximum mass range
Not Recorded	Not applicable	Not Applicable	Mid-range mass of female and male lightest and heaviest limits combined

**Table 4:** Total number of cats (target) and by-catch (non-target) trapped at each site.

Site	Target	Non-Target
Boyicup	0	11
Balban	0	10
Perup Sanctuary	0	8
Mt Gibson Sanctuary	2	42
Mornington Sanctuary	19	7
Francois Peron National Park	3455	353

**Table 5:** The number of individuals captured at Francois Peron National Park from each taxon when different lure types were used. Percentage deviations (a measure of the extent to which the observed chi-square value varies from that expected if there is no association between the variables) are shown for each result.

Taxon	Olfactory Lure	Other Lure
Bird	18 (+16.9%)	24 (−9.8%)
Mammal	28 (−25.1%)	74 (+14.6%)
Reptile	20 (+51.5%)	16 (−29.8%)

**Table 6:** Number of by-catch individuals (native and non-native species) in each category (uninjured, intermediate, and severe). Percentage deviations (a measure of the extent to which the observed chi-square value varies from that expected if there is no association between the variables) are shown.

Injury Category	Native	Non-Native
Uninjured	167 (−7.7%)	169 (+8.9%)
Intermediate	22 (+57.2%)	4 (−66.7%)
Severe	43 (+15.8%)	26 (−18.4%)
Total	232	199

**Table 7:** Number of individuals from all three taxa of native by-catch (bird, mammal and reptile) in each of the summarised injury categories (uninjured, intermediate and severe). Percentage deviation is also displayed next to the corresponding value.

Injury Category	Bird	Mammal	Reptile
Uninjured	32 (−14.5%)	107 (+7.7%)	28 (−7.4%)
Intermediate	3 (−39.2%)	14 (+7%)	5 (+25.5%)
Severe	17 (+76.4%)	17 (−33.5%)	9 (+15.6%)
Total	52	138	42

**Table 8:** Mean mass ± standard deviation (kg) of all three taxa of native by-catch (bird, mammal and reptile) in each of the summarised injury categories (uninjured, intermediate and severe).

Injury Category	Bird	Mammal	Reptile
Uninjured	14.83 (18.43)	9.19 (13.72)	1.07 (0.45)
Intermediate	0.82 (0.72)	6.94 (10.07)	1.38 (0.24)
Severe	0.29 (0.16)	5.02 (11.64)	0.91 (0.42)
